# Maladie du Paget du mamelon

**DOI:** 10.11604/pamj.2017.26.65.8463

**Published:** 2017-02-03

**Authors:** Saoussane Kharmoum, Rabie Rahhali

**Affiliations:** 1 Service d’Oncologie, centre hospitalier provincial Duc de Tovar, Tanger, Maroc

**Keywords:** Maladie du Paget, mamelon, carcinome canalaire in situ, Paget’s disease, nipple, ductal carcinoma in situ

## Image en médecine

Nous rapportons le cas d’une patiente âgée de 53 ans, ménopausée, sans antécédents pathologiques particuliers, qui présente depuis une année une lésion squameuse et prurigineuse de la plaque aréolo-mamelonnaire droite (A), l’examen clinique ne révèle pas de masses mammaires palpables, les aires ganglionnaires sont libres. La mammographie et l’échographie mammaire trouvent un épaississement du revêtement cutané péri-aréolaire droit sans lésions suspecte sous-jacente. Une biopsie a été réalisé objectivant un épiderme papillomateux pénétré par de nombreuses cellules tumorales malignes type Paget (B), l’immunohistochimie a montré des cellules tumorales positives pour la cytokératine CK7 et négatives pour la CK20, avec une surexpression de l’HER2. Au vu de ces résultats le diagnostic de maladie de Paget a été retenu. La maladie de Paget correspond à l’envahissement de l’épiderme mamelonnaire par un carcinome canalaire in situ (CIS), il s’agit d’une variante rare du CIS, elle représente 1 à 3 % des tumeurs mammaires. Elle peut être associée à une néoplasie mammaire dans plus de 80% des cas, d’où l’intérêt de réaliser un bilan sénologique complet. La maladie de Paget doit être suspectée et recherchée devant toute lésion unilatérale et persistante du mamelon. Le traitement dépend essentiellement de la présence ou non d’un cancer mammaire sous-jacent. Il consiste à réaliser une mastectomie ou une chirurgie conservatrice du sein avec un curage ganglionnaire. Les traitements adjuvants (radiothérapie, chimiothérapie, immunothérapie et hormonothérapie) doivent être discutés au cas par cas.

**Figure 1:**
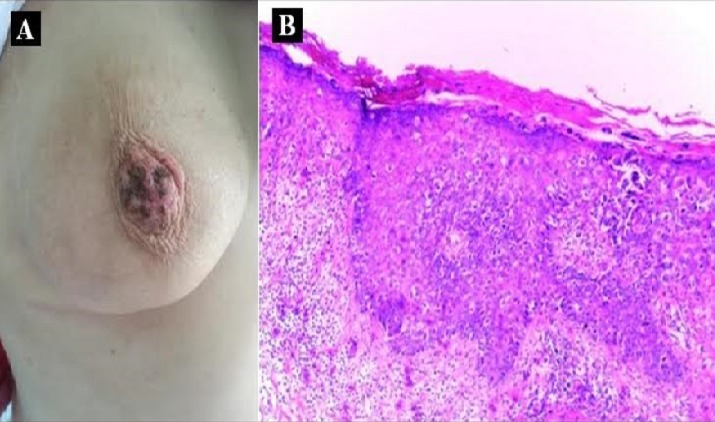
A) lésion squameuse et prurigineuse de la plaque aréolo-mamelonaoire droite; B) infiltration pagetoide de l’épiderme par des cellules de grande taille à cytoplasme clair, atypiques, isolées ou groupées en amas (hématoxiline, eosine x20)

